# Tricaesium citrate monohydrate, Cs_3_C_6_H_5_O_7_·H_2_O: crystal structure and DFT comparison

**DOI:** 10.1107/S205698901700367X

**Published:** 2017-03-14

**Authors:** Alagappa Rammohan, Amy A. Sarjeant, James A. Kaduk

**Affiliations:** a Atlantic International University, Honolulu, HI, USA; b Department of Chemistry, Northwestern University, Evanston, IL, USA; c Illinois Institute of Technology, Department of Chemistry, 3101 S. Dearborn St., Chicago, IL 60616, USA

**Keywords:** crystal structure, density functional theory, citrate, caesium

## Abstract

The crystal structure of tricaesium citrate monohydrate, 3Cs^+^·C_6_H_5_O_7_^3−^·H_2_O, has been solved and refined using laboratory X-ray single-crystal diffraction data, and optimized using density functional techniques. This compound is isostructural to the K^+^ and Rb^+^ compounds with the same formula. The three independent Cs cations are eight-, eight-, and seven-coordinate, with bond-valence sums of 0.91, 1.22, and 1.12 valence units. The coordination polyhedra link into a three-dimensional framework. The hy­droxy group forms the usual *S*(5) hydrogen bond with the central carboxyl­ate group, and the water mol­ecule acts as a donor in two strong hydrogen bonds.

## Abstract 2

The crystal structure of tricaesium citrate monohydrate has been solved and refined using laboratory X-ray single-crystal diffraction data, and optimized using density functional techniques.

## Chemical context   

In the course of a systematic study of the crystal structures of Group 1 (alkali metal) citrate salts to understand the anion’s conformational flexibility, ionization, coordination tendencies, and hydrogen bonding, we have determined several new crystal structures. Most of the new structures were solved using powder diffraction data (laboratory and/or synchrotron), but single crystals were used where available. The general trends and conclusions about the sixteen new compounds and twelve previously characterized structures are being reported separately (Rammohan & Kaduk, 2017*a* ▸). Twelve of the new structures – NaKHC_6_H_5_O_7_, NaK_2_C_6_H_5_O_7_, Na_3_C_6_H_5_O_7_, NaH_2_C_6_H_5_O_7_, Na_2_HC_6_H_5_O_7_, K_3_C_6_H_5_O_7_, Rb_2_HC_6_H_5_O_7_, Rb_3_C_6_H_5_O_7_·H_2_O, Rb_3_C_6_H_5_O_7_, Na_5_H(C_6_H_5_O_7_)_2_, CsH_2_C_6_H_5_O_7_, and Cs_2_HC_6_H_5_O_7_ – have been published recently (Rammohan & Kaduk, 2016*a* ▸,*b* ▸,*c* ▸,*d* ▸,*e* ▸, 2017*b* ▸,*c* ▸,*d* ▸,*e* ▸,*f* ▸; Rammohan *et al.*, 2016 ▸, 2017 ▸), and three additional structures – KH_2_C_6_H_5_O_7_ KH_2_C_6_H_5_O_7_·H_2_O_2_, and Cs_3_C_6_H_5_O_7_ – have been communicated to the CSD (Kaduk & Stern, 2016*a* ▸,*b* ▸; Rammohan & Kaduk, 2017*g* ▸).
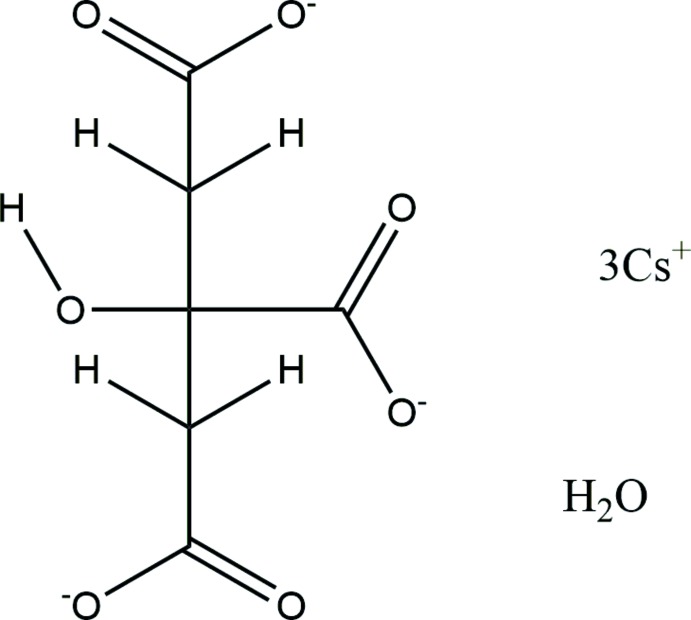


## Structural commentary   

**Figure 1:**
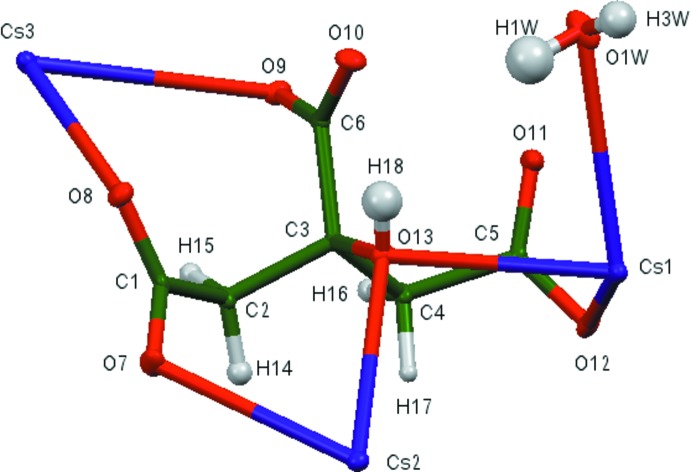
The asymmetric unit of the title compound, with the atom numbering. The atoms are represented by 50% probability displacement ellipsoids.

**Figure 2:**
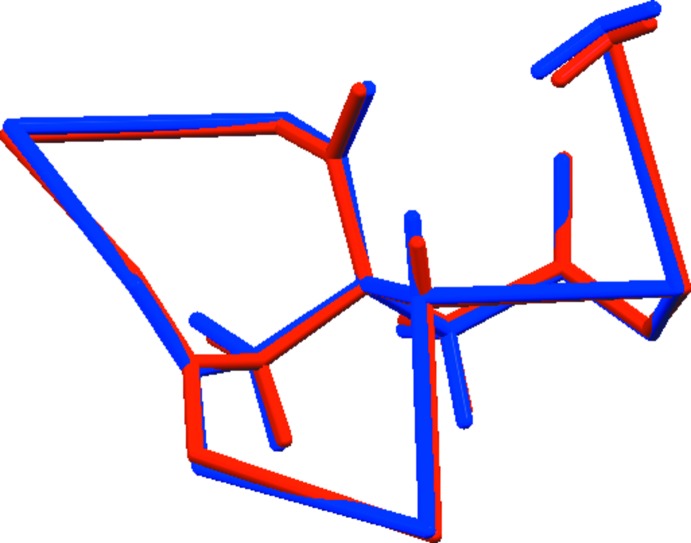
Comparison of the refined and optimized structures of tricaesium citrate monohydrate. The refined structure is in red and the DFT-optimized structure is in blue.

The asymmetric unit of the title compound is shown in Fig. 1 ▸. The root-mean-square deviation of the non-hydrogen atoms in the experimental and DFT-optimized structures is 0.123 Å (Fig. 2 ▸). The largest difference is 0.200 Å, at O1*W*. This good agreement provides strong evidence that the experimental structure is correct (van de Streek & Neumann, 2014 ▸). Almost all of the bond lengths, bond angles, and torsion angles in the experimentally determined structure fall within the normal ranges indicated by a *Mercury* Mogul geometry check (Macrae *et al.*, 2008 ▸). Only the O8—C1—C2 angle of 118.0° is flagged as unusual [average = 119.8 (4)°, Z-score = 4.2). The Z-score is the result of the exceptionally low uncertainty on the average of this bond angle. In the DFT-optimized structure, the O7—C1—C2 angle of 115.9° is flagged as unusual [average = 120.3 (12)°, Z-score = 3.6]. The citrate anion occurs in the *trans,trans* conformation, which is one of the two low-energy conformations of an isolated citrate. The three Cs^+^ cations are eight-, eight-, and seven-coordinate, with bond-valence sums of 0.91, 1.22, and 1.12 valence units. There is extensive chelation of the citrate anion to Cs^+^ cations: O12(end)/O13(OH) to Cs1, O8(end)/O10(central) to Cs2, O11(end)/O10(central) to Cs2, C11(end)/O9(central) to Cs2, O7(end)/O13(OH) to Cs2, O8(end)/O9(central) to Cs3, and O11(end)/O11(central) to Cs3. The carboxyl­ate group O11/O12 also acts as a bidentate ligand to Cs1. The Mulliken overlap populations and atomic charges indicate that the metal–oxygen bonding is ionic.

The BFDH (Bravais–Friedel–Donnay–Harker) morph­ology (Bravais, 1866 ▸; Friedel, 1907 ▸; Donnay & Harker, 1937 ▸) is blocky, with {011} as major faces. The powder pattern exhibited strong preferred orientation consistent with {101} as the major faces of plates. These faces are also significant in the BFDH morphology.

## Supra­molecular features   

**Figure 3:**
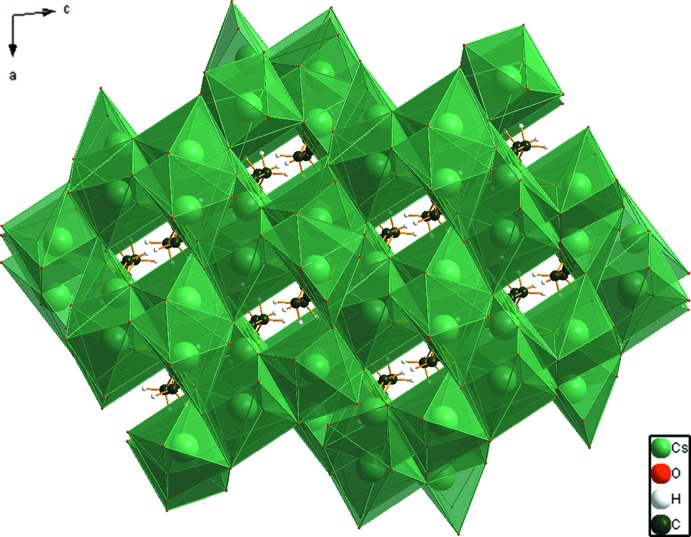
Crystal structure of Cs_3_C_6_H_5_O_7_·H_2_O, viewed down the *b* axis.

The coordination polyhedra link into a three-dimensional framework (Fig. 3 ▸). The hydro­phobic methyl­ene groups occupy pockets in the framework. The hy­droxy group forms the usual *S*(5) hydrogen bond with the central carboxyl­ate group, and the water mol­ecule acts as a donor in two strong hydrogen bonds (2.686 and 2.662 Å). By the correlation between the square root of the Mulliken overlap population and hydrogen-bond energy derived in Rammohan & Kaduk (2017*a* ▸), these hydrogen bonds contribute 14.4, 14.1, and 14.1 kcal mol^−1^, respectively, to the crystal energy. Numerical details of the hydrogen bonds in the experimentally determined and DFT-optimized structures are given in Tables 1 ▸ and 2 ▸, respectively.

## Database survey   

Details of the comprehensive literature search for citrate structures are presented in Rammohan & Kaduk (2017*a* ▸). A reduced-cell search of the cell of tricaesium citrate monohydrate in the Cambridge Structural Database (Groom *et al.*, 2016 ▸) (increasing the default tolerance from 1.5 to 2.0%) yielded 258 hits, but combining the cell search with the elements C, H, Cs, and O only yielded no hits. Increasing the tolerance to 5% with C, H, O, and Rb only yielded trirubidium citrate monohydrate (Love & Patterson, 1960 ▸; CSD refcode ZZZHZC), but no coordinates were reported for this phase. The structure has since been reported by Rammohan & Kaduk (2017*c* ▸). Increasing the tolerance on the cell to 7% with C, H, K, and O only yielded K_3_C_6_H_5_O_7_·H_2_O (Burns & Iball, 1954 ▸, CSD refcode ZZZHVI; Carrell *et al.*, 1987 ▸, CSD refcodes ZZZHVI01 and ZZZHVI02). This compound is isostructural to the K^+^ (Carrell *et al.*, 1987 ▸) and Rb^+^ (Rammohan & Kaduk, 2017*c* ▸) compounds with the same formula, but the previously-reported structure of K_3_C_6_H_5_O_7_·H_2_O has to be transformed from setting *P*2_1_ */a* to *P*2_1_ */n* to make the similarities clear (Table 3 ▸).

## Synthesis and crystallization   

H_3_C_6_H_5_O_7_·H_2_O (2.0774 g, 10.0 mmol, Sigma–Aldrich) was dissolved in 8 ml deionized water. Cs_2_CO_3_ (4.9324 g, 15.1 mmole, Sigma–Aldrich) was added to the citric acid solution slowly with stirring. The resulting clear colorless solution was evaporated to dryness in a oven at 333 K. Single crystals were isolated from the white product.

## Refinement   

Crystal data, data collection and structure refinement details are summarized in Table 4 ▸. The hydrogen atoms were freely refined with isotropic ADPs. The lattice parameters at 300 K (derived from a Le Bail fit of the powder pattern) are *a* = 7.8851 (4), *b* = 12.2109 (8), *c* = 14.0367 (8) Å, β = 97.280 (4)°, and *V* = 1340.63 (14) Å^3^.

## DFT calculations   

After the Rietveld refinement, a density functional geometry optimization (fixed experimental unit cell at 100 K) was carried out using *CRYSTAL09* (Dovesi *et al.*, 2005 ▸). The basis sets for the C, H, and O atoms were those of Gatti *et al.* (1994 ▸), and the basis set for Cs was that of Prencipe (1990 ▸). The calculation used 8 *k*-points and the B3LYP functional, and took about 85 h on a 2.4 GHz PC. The *U_eq_* values from the refinement were assigned to the optimized fractional coordinates.

## Supplementary Material

Crystal structure: contains datablock(s) I, I_DFT. DOI: 10.1107/S205698901700367X/vn2126sup1.cif

Structure factors: contains datablock(s) I. DOI: 10.1107/S205698901700367X/vn2126Isup2.hkl

Supporting information file. DOI: 10.1107/S205698901700367X/vn2126Isup3.cml

CCDC reference: 1536526

Additional supporting information:  crystallographic information; 3D view; checkCIF report

## Figures and Tables

**Table 1:** Hydrogen-bond geometry (Å, °) for (I)

*D*—H⋯*A*	*D*—H	H⋯*A*	*D*⋯*A*	*D*—H⋯*A*
O13—H13⋯O10	0.75 (5)	2.06 (5)	2.579 (2)	127 (4)
O1*W*—H1*WA*⋯O7^i^	0.88 (5)	1.81 (5)	2.684 (3)	174 (5)
O1*W*—H1*WB*⋯O9^ii^	0.76 (5)	1.95 (5)	2.695 (3)	167 (4)

Symmetry codes: (i) 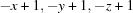; (ii) 
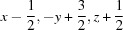
.

**Table 2:** Hydrogen-bond geometry (Å, °) for the DFT-optimized structure[Chem scheme1]

*D*—H⋯*A*	*D*—H	H⋯*A*	*D*⋯*A*	*D*—H⋯*A*
O13—H18⋯O10	0.984	1.811	2.538	127.9
O1*W*—H1*W*⋯O7^i^	0.984	1.719	2.686	166.8
O1*W*—H3*W*⋯O9^ii^	0.981	1.697	2.662	167.0

Symmetry codes: (i) 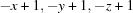; (ii) 
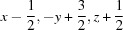
.

**Table 3:** Lattice parameters (Å, °, Å^3^, K; space group *P*2_1_/*n*) of *M*_3_C_6_H_5_O_7_·H_2_O

	K*^*a*^*	Rb*^*b*^*	Cs*^*c*^*
*a*	7.092 (2)	7.4477 (1)	7.88551 (4)
*b*	11.772 (1)	11.8755 (2)	12.2109 (8)
*c*	12.865 (1)	13.4167 (2)	14.0367 (8)
β	98.031 (2)	97.8820 (9)	97.280 (4)
*V*	1063.50	1175.44 (3)	1340.63 (14)
*V*/non-H	16.6	17.3	19.7
*T*	300	300	100

References: (*a*) Carrell *et al.* (1987 ▸); (*b*) Rammohan & Kaduk (2017*c* ▸); (*c*) this work.

**Table 4:** Experimental details

	X-ray data
Crystal data	
Chemical formula	3Cs^+^·C_6_H_5_O_7_^3−^·H_2_O
*M*_r_	605.85
Crystal system, space group	Monoclinic, *P*2_1_/*n*
Temperature (K)	100
*a*, *b*, *c* (Å)	7.7529 (3), 12.0281 (4), 13.8043 (5)
β (°)	97.000 (2)
*V* (Å^3^)	1277.69 (8)
*Z*	4
Radiation type	Mo *K*α
μ (mm^−1^)	8.54
Crystal size (mm)	0.37 × 0.28 × 0.20

Data collection	
Diffractometer	Bruker Kappa APEX CCD area detector
Absorption correction	Multi-scan (*SADABS*; Bruker, 2008 ▸)
*T*_min_, *T*_max_	0.485, 0.747
No. of measured, independent and observed [*I* > 2σ(*I*)] reflections	33171, 6191, 5701
*R*_int_	0.040
(sin θ/λ)_max_ (Å^−1^)	0.834

Refinement	
*R*[*F* ^2^ > 2σ(*F* ^2^)], *wR*(*F* ^2^), *S*	0.024, 0.047, 1.16
No. of reflections	6191
No. of parameters	183
H-atom treatment	All H-atom parameters refined
Δρ_max_, Δρ_min_ (e Å^−3^)	1.41, −1.19

The same symmetry and lattice parameters were used for the DFT calculations. Computer programs: *APEX2* and *SAINT* (Bruker, 2008 ▸), *XM* (Sheldrick, 2008 ▸), *SHELXL2014* (Sheldrick, 2015 ▸) and *OLEX2* (Dolomanov *et al.*, 2009 ▸).

## supplementary crystallographic information

### Crystal data

**Table:**

3Cs^+^·C_6_H_5_O_7_^3^^−^·H_2_O	β = 97.0000°
*M**_r_* = 605.85	*V* = 1277.69 Å^3^
Monoclinic, *P*2_1_/*n*	*Z* = 4
*a* = 7.7529 Å	DFT calculation radiation, λ = 1.54184 Å
*b* = 12.0281 Å	*T* = 100 K
*c* = 13.8043 Å	

### Data collection

**Table:**

*h* = →	*l* = →
*k* = →	

### Refinement

**Table:**

H-atom parameters not refined	

### Fractional atomic coordinates and isotropic or equivalent isotropic displacement parameters (Å^2^)

**Table:**

	*x*	*y*	*z*	*U*_iso_*/*U*_eq_	
Cs1	0.50780	0.82708	0.61694	0.00960*	
Cs2	0.83209	0.55810	0.61104	0.00867*	
Cs3	0.38395	0.43445	0.11206	0.01077*	
O7	0.78685	0.43530	0.42471	0.01460*	
O8	0.53202	0.41899	0.32856	0.01190*	
O9	0.40939	0.69538	0.19097	0.01090*	
O10	0.28355	0.67716	0.32774	0.01250*	
O11	0.46920	0.91349	0.32953	0.01330*	
O12	0.71297	0.95547	0.42842	0.01600*	
O13	0.56194	0.66231	0.44450	0.00980*	
H18	0.43476	0.65343	0.43651	0.02800*	
C1	0.67041	0.46915	0.35846	0.00830*	
C2	0.70544	0.58130	0.31182	0.00870*	
H14	0.84281	0.60200	0.33092	0.01100*	
H15	0.68245	0.57527	0.23228	0.00900*	
C3	0.59503	0.67849	0.34578	0.00720*	
C4	0.70484	0.78402	0.34170	0.00980*	
H16	0.74216	0.79325	0.26803	0.01000*	
H17	0.82267	0.77093	0.39247	0.00600*	
C5	0.62112	0.89281	0.36933	0.00800*	
C6	0.41465	0.68598	0.28244	0.00790*	
O1W	0.09706	0.77363	0.54410	0.01850*	
H1W	0.14970	0.69912	0.54760	0.04000*	
H3W	0.04287	0.78150	0.60432	0.02400*	

### Bond lengths (Å)

**Table:**

O7—C1	1.271	C2—H15	1.093
O8—C1	1.256	C2—C3	1.555
O9—C6	1.263	C3—C4	1.533
O10—C6	1.261	C3—C6	1.559
O11—C5	1.262	C4—H16	1.096
O12—C5	1.264	C4—H17	1.093
O13—H18	0.984	C4—C5	1.529
O13—C3	1.430	O1W—H1W	0.984
C1—C2	1.533	O1W—H3W	0.981
C2—H14	1.094		

### Hydrogen-bond geometry (Å, º)

**Table:**

*D*—H···*A*	*D*—H	H···*A*	*D*···*A*	*D*—H···*A*
O13—H18···O10	0.984	1.811	2.538	127.9
O1*W*—H1*W*···O7^i^	0.984	1.719	2.686	166.8
O1*W*—H3*W*···O9^ii^	0.981	1.697	2.662	167.0

Symmetry codes: (i) −*x*+1, −*y*+1, −*z*+1; (ii) *x*−1/2, −*y*+3/2, *z*+1/2.
